# Mouse resources at the RIKEN BioResource Research Center and the National BioResource Project core facility in Japan

**DOI:** 10.1007/s00335-021-09916-x

**Published:** 2021-09-16

**Authors:** Saori Mizuno-Iijima, Toshiaki Nakashiba, Shinya Ayabe, Hatsumi Nakata, Fumio Ike, Noriko Hiraiwa, Keiji Mochida, Atsuo Ogura, Hiroshi Masuya, Shoko Kawamoto, Masaru Tamura, Yuichi Obata, Toshihiko Shiroishi, Atsushi Yoshiki

**Affiliations:** 1 Experimental Animal Division, RIKEN BioResource Research Center, Tsukuba, Ibaraki 305-0074 Japan; 2 Bioresource Engineering Division, RIKEN BioResource Research Center, Tsukuba, Ibaraki 305-0074 Japan; 3 Integrated Bioresource Information Division, RIKEN BioResource Research Center, Tsukuba, Ibaraki 305-0074 Japan; 4 Genetics Informatics Laboratory, National Institute of Genetics, Mishima, 411-8540 Japan; 5 Technology and Development Team for Mouse Phenotype Analysis, RIKEN BioResource Research Center, Tsukuba, Ibaraki 305-0074 Japan; 6 RIKEN BioResource Research Center, Tsukuba, Ibaraki 305-0074 Japan

## Abstract

The RIKEN BioResource Research Center (BRC) was established in 2001 as a comprehensive biological resource center in Japan. The Experimental Animal Division, one of the BRC infrastructure divisions, has been designated as the core facility for mouse resources within the National BioResource Project (NBRP) by the Japanese government since FY2002. Our activities regarding the collection, preservation, quality control, and distribution of mouse resources have been supported by the research community, including evaluations and guidance on advancing social and research needs, as well as the operations and future direction of the BRC. Expenditure for collection, preservation, and quality-control operations of the BRC, as a national core facility, has been funded by the government, while distribution has been separately funded by users’ reimbursement fees. We have collected over 9000 strains created mainly by Japanese scientists including Nobel laureates and researchers in cutting-edge fields and distributed mice to 7000 scientists with 1500 organizations in Japan and globally. Our users have published 1000 outstanding papers and a few dozen patents. The collected mouse resources are accessible via the RIKEN BRC website, with a revised version of the searchable online catalog. In addition, to enhance the visibility of useful strains, we have launched web corners designated as the “Mouse of the Month” and “Today’s Tool and Model.” Only high-demand strains are maintained in live colonies, while other strains are cryopreserved as embryos or sperm to achieve cost-effective management. Since 2007, the RIKEN BRC has built up a back-up facility in the RIKEN Harima branch to protect the deposited strains from disasters. Our mice have been distributed with high quality through the application of strict microbial and genetic quality control programs that cover a globally accepted pathogens list and mutated alleles generated by various methods. Added value features, such as information about users’ publications, standardized phenotyping data, and genome sequences of the collected strains, are important to facilitate the use of our resources. We have added and disseminated such information in collaboration with the NBRP Information Center and the NBRP Genome Information Upgrading Program. The RIKEN BRC has participated in international mouse resource networks such as the International Mouse Strain Resource, International Mouse Phenotyping Consortium, and Asian Mouse Mutagenesis and Resource Association to facilitate the worldwide use of high-quality mouse resources, and as a consequence it contributes to reproducible life science studies and innovation around the globe.

## Foundation of the RIKEN BioResource Research Center

The RIKEN BioResource Research Center (BRC) was established in January 2001 (https://web.brc.riken.jp/en/about). During these past 20 years, under the leadership of Dr. Toshihiko Shiroishi (current Director), Dr. Yuichi Obata (former Director and current senior adviser), and the late Dr. Kazuo Moriwaki (founding Director), the RIKEN BRC has successfully established its operational structure as a global biological resource center. The RIKEN BRC has been operating since its foundation according to three principles: trust, sustainability, and leadership. Our activities have been strongly supported by the scientific community as well as by the Ministry of Education, Culture, Sports, Science and Technology (MEXT) of the Japanese government and RIKEN, the founding institution. Our collection, preservation, and quality-control (QC) operations have been funded by the government, while the distribution service has been funded separately by income generated by reimbursement fees collected from users.

To solve problems in the 21st Century related to health, food, the environment, and sustainable development, the importance of bioresources has grown exponentially. Among the many bioresources used in life science and biotechnology, the RIKEN BRC has specifically focused on the mouse, the most important experimental animal; Arabidopsis, the most important experimental plants; cultured cell lines of humans and animals; genetic materials from humans, animals, and microorganisms; and microorganisms such as bacteria, fungi, and archaea. These biological resources have been chosen according to criteria such as their importance to Japanese society and science, their original creation or derivation by Japanese scientists, the presence of a critical mass of users, and their merits for large-scale and intensive management. The RIKEN BRC has actively collected, preserved, conducted QC of, and distributed these biological resources with associated information and played a crucial role as a comprehensive biological resource hub to promote the life sciences and innovation. The RIKEN BRC has earned its status as one of the state-of-the-art research infrastructure centers of RIKEN.

## Participation in the National BioResource Project

The National BioResource Project (NBRP) was launched by MEXT in FY2002, one year after the foundation of the RIKEN BRC (Yamazaki et al. 2010; Yokoyama et al. 2010; Yoshiki et al. 2009). The main program of the NBRP is the Core Facility Upgrading Program which aims to collect, preserve, and provide essential bioresources for life science studies strategically and systematically by the national government. Four divisions of the RIKEN BRC were selected at the start of the program as national core facilities of respective bioresources including the Experimental Animal Division as the NBRP core facility for mice (Yoshiki et al. 2009). Our activities as a national core facility have been evaluated extensively by the NBRP evaluation committee and designated over the course for 20 years during four 5-year programs.

## Meeting social and research needs

Communication with the scientific community and comprehension of its advancing needs are essential to execute the mission of the research infrastructure. To achieve this objective, the RIKEN BRC has asked the domestic Resource Committee annually and the international RIKEN BRC Advisory Council (BRAC) every two or three years, each composed of several leading scientists and specialists in the biomedical sciences and global research infrastructures, for advice and recommendations regarding research needs and future directions. Moreover, the RIKEN Advisory Council provides recommendations on general research activities and management of the entire RIKEN institute, including the BRC, for guiding future research strategies and improving management structures. These recommendations form an integral part of the sustainable operation of the RIKEN BRC as a key element of global research infrastructure. The reports of previous Resource Committee and BRAC meetings are posted on the RIKEN BRC website (https://web.brc.riken.jp/en/reports) to ensure transparent operation. To earn trust in our activities for mouse resources, the RIKEN BRC has been supported by the Animal Experiments Committee and Recombinant DNA Committee which involve external scientists and non-scientists and include representatives of local residents and the municipal office of the city of Tsukuba to ensure animal welfare and safety measures in recombinant DNA experiments.

## Collection of mouse strains developed in Japan

Mice are the premier mammalian model organism in terms of the availability of genetically defined inbred strains, high-quality genome information, advanced technologies for genomic alterations and phenotyping, and accumulated knowledge about their genetic diseases (Lloyd et al. 2020). Such model mammals are indispensable in an aging society and to achieve precision medicine by serving tools used to elucidate the molecular mechanisms of the onset of diseases such as cancer, dementia, and lifestyle-related diseases and to develop therapies as well. The Experimental Animal Division of the RIKEN BRC has collected useful mouse strains mainly developed by Japanese scientists to ensure that the collection does not overlap with those of other repositories around the world (Yoshiki et al. 2009).

**Fig. 1:**
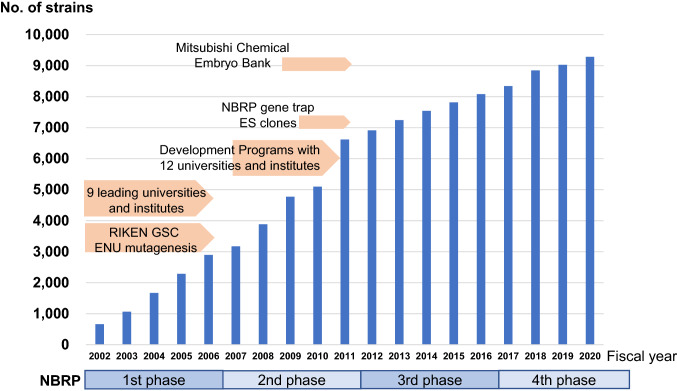
Number of mouse resources archived at the RIKEN BRC/NBRP

In the first phase of the NBRP, we collaborated with a large-scale ethylnitrosourea (ENU) mutagenesis project conducted by the Genome Science Center of RIKEN (Sakuraba et al. 2005) in addition to nine leading domestic universities and institutes. The collection has grown rapidly through collaborating facilities, with over 500 new strains added per year (Fig. 1). In the second phase of the NBRP, the archive expanded even more rapidly through the deposition of a large collection of frozen embryos from the Embryo Bank of the Mitsubishi Chemical Company and the gene-trap embryonic stem cell resources created by Dr. Yasumasa Ishida in the NBRP Fundamental Technology Upgrading Program (Shigeoka et al. 2005), as well as collaborative development programs for advanced genetically modified mouse models with 12 universities and institutes in Japan (Fig. 1). We have continued to ask individual scientists who have developed and published novel genetically modified strains to deposit their mice at the RIKEN BRC. In the third through fourth phases of the NBRP, we have focused on genetically modified strains to achieve an understanding of higher-order biological phenomena and advance research and development for overcoming diseases. Recently, genome-edited knockout and knock-in mice created using CRISPR/Cas9 have gradually accumulated in the collection since FY2014 and overtaken ES cell-based genetically modified mice since FY2019. The collection has steadily increased with the deposition of about 300 strains from 50 scientists on average each year, leading to the archiving of over 9,000 mouse models as of March 2021 (Fig. 1).

**Fig. 2:**
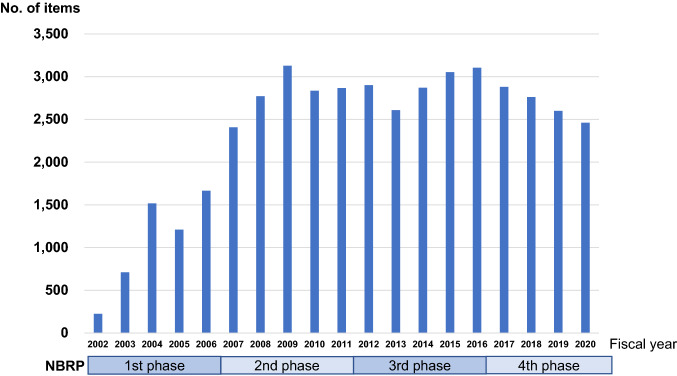
Number of mouse resource items distributed by the RIKEN BRC/NBRP

## High-demand and high-impact strains

Our collection includes various gene knockouts, fluorescent reporters, conditional strains containing Cre–lox and Flp–FRT systems, and other useful congenic and inbred strains. Several mouse strains developed by Nobel laureates have also been deposited by the laureates and their colleagues, such as the stem cell reporter Nanog-EGFP knock-in mouse (RBRC02290) developed by Dr. Shinya Yamanaka (Okita et al. 2007), the autophagy reporter GFP-LC3#53 transgenic mouse (RBRC00806) developed by Dr. Noboru Mizushima (Kuma et al. 2004), and the PD-1 knockout mouse strain (RBRC02142) developed by Dr. Tasuku Honjo (Nishimura et al. 1999). Within the collection, there are a dozen unique strains with long-term periods of high demand that have been utilized in hundreds of publications by users (Table 1). These user publications have subsequently been cited in an outstanding number of studies (Table 1), indicating the large impact on the relevant research community.

**Table 1:** Number of user publications and citations associated with research using mouse strains distributed by the RIKEN BRC

RBRC no	Strain name	Category: description	No. of publications	Total no. of citations
00806	C57BL/6-Tg(CAG-EGFP/LC3)53NmzRbrc	TG: GFP-tagged reporter for autophagosome LC3	108	**18,891**
00267	C57BL/6-Tg(CAG-EGFP)C14-Y01-FM131OsbRbrc	TG: Green mice, EGFP expression in all tissues	53	2484
01390	B6.129P2-*Nfe2l2*^*tm1Mym*^/MymRbrc	Null-KO: *Nfe2l2*, nuclear factor, erythroid derived 2, like 2 gene null deficient mice	46	2277
01834	C57BL/6-Tg(CAG-flpe)36Ito/ItoRbrc	TG: Ubiquitous deleter for Flp-FRT recombination system	30	1092
00858	B6;129S6- *Bcl2l12/Irf3*^*tm1Ttg*^/TtgRbrc	Null-KO: Interferon regulatory factor 3 gene	24	964
01420	B6;129P2-*Irf7*^*tm1Ttg*^/TtgRbrc	Null-KO: Interferon regulatory factor 7 gene, exons 2–3 replaced by the neo cassette	23	888
01828	B6.Cg-Tg(CAG-Cre)CZ-MO2OsbRbrc	TG: Ubiquitous deleter for Cre-loxP recombination system	23	657
02975	B6.129S-*Atg5*^*tm1Myok*^/Rbrc	cKO: Autophagy-related 5 gene floxed knockout mouse	23	**4576**
01361	B6.Cg-*Trp53*^*tm1Sia*^/Rbrc	Null-KO: Cancer related p53 deficient mice	21	275
00144	C57BL/6-*Ly5.1*/Rbrc	B6 congenic strain carrying Ly5.1: Useful for tissue/cell and B cell markers in transplantation studies	19	371
00209	MSM/MsRbrc	Japanese wild-derived inbred	19	577
00639	JF1/MsRbrc	Japanese wild-derived inbred	18	363

Two autophagy-related strains, RBRC00806 and 02975 which are given in bold showed highest impact in regard to publications with out standing number of citations

*TG* transgenic mouse, *KO*; knockout mouse, *KI* knock-in mouse, *cKO* conditional knockout mouse

It is very important for the resource repository to continuously collect novel strains created in cutting-edge research for sustainable operations to meet the needs of advancing life science research and innovation. Notably, the C57BL/6-*App*^*tm3(NL−G−F)Tcs*^/TcsRbrc (RBRC06344) strain, a next-generation Alzheimer's disease (AD) model created by Dr. Takaomi C. Saido and his colleagues at the RIKEN Center for Brain Science, has become the strain with the highest demand since FY2015 soon after its deposition. This novel AD knock-in model contains humanized sequences and clinical mutations in the endogenous mouse *App* gene, exhibiting typical Aβ pathology, neuroinflammation, and memory impairment in an age-dependent manner (Saito et al. 2014; Sasaguri et al. 2017). The next-generation AD model has been further advanced by crossing the model strain with human *MAPT* knock-in mice (Saito et al. 2019) for future preclinical studies.

## Accessibility and distribution

Mouse strains deposited at the RIKEN BRC can be accessed via the BRC home page (https://mus.brc.riken.jp/en/), or browsed or searched from the web catalog (https://mus.brc.riken.jp/en/search_for_mouse_strain). Mice are distributed in the form of live animals, frozen embryos or sperm, and recovered litters from frozen embryos or sperm. Organs, tissues, and genomic DNA have been provided on request. We use a material transfer agreement to protect the intellectual property (IP) right of the depositor and other IP rights associated with the strain, while clarifying for users the terms and conditions set forth by the depositor. The total distribution increased in proportion to the size of the collection during the first phase of the NBRP. Through the second to fourth phases of the NBRP, 2500–3000 items of mouse resources per year were stably distributed (Fig. 2). We have so far distributed our mouse resources to 4377 users at 627 domestic organizations and 3225 users at 917 overseas organizations in 41 countries, resulting in 1047 user publications and 41 patents. Among these resources, autophagy reporter GFP-LC3 mice (RBRC00806) have been used since FY2005 at 272 organizations in 24 countries, resulting in 108 publications. Since FY2015, the knock-in AD model has been requested most frequently and distributed to 175 organizations in 16 countries.

## Dissemination of information

Dissemination of information associated with mouse strains has a vital role in facilitating active use of large-scale mouse resources by the biomedical community. We have recently renewed the RIKEN BRC home page and developed a revised version of the searchable online catalog of mouse strains that functions as a part of the BRC-integrated database (Masuya et al. 2021).

Monthly email newsletters are sent to announce newly deposited mouse strains, users’ publications, relevant news, and coming events in both Japanese and English. To complement the functions of the integrated database and email news, we have launched proactive outreach corners focusing on specific strains, such as “*Today’s Tool for Functional Analysis,” “Today’s Model for Human Disease” and* (https://mus.brc.riken.jp/en/todays_tool_and_model), and *“Mouse of the Month”* (https://mus.brc.riken.jp/en/mouse_of_month) to provide researchers with information about advanced strains and facilitate the use of such strains for cutting-edge research. For the Mouse of the Month, strains are selected based on the potential impact of relevant publications as well as access log data for strains from the web catalog. The articles are designed in a mini-review format that includes representative images or figures and a summary of the strain including gene details, use applications, the latest research results, and references. The strain name with its repository unique identifier (RBRC number) is linked to each strain data sheet page of the online catalog supported by the BRC-integrated database. The Today's Tool and Model series are presented in a list format to disseminate more genetic tools and disease models; the list provides the title of the strain, its strain ID with a link to its strain data sheet, brief descriptions, and references with PubMed links.

## Cryopreservation and secure back-up storage

Mice with high demand are maintained as live stocks for faster distribution, while mice with lower demand are preserved as frozen sperm or embryos in liquid nitrogen tanks for cost-effective management. Two-cell stage embryos of various strains have been successfully cryopreserved by using ethylene glycol-based vitrification (Mochida et al. 2011). Sperm samples were cryopreserved by the method reported by Nakagata and Takeshima (1993). Recent advances in assisted reproductive technologies have facilitated efficient preservation and recovery of various inbred strains and dozens of unique wild-derived strains belonging to different species and subspecies of *Mus* (Hasegawa et al. 2012, 2016, 2021; Mochida et al. 2014). Cryopreservation also plays a key role in protecting the deposited mouse strains by minimizing genetic drift, which may occur during continuous breeding periods (Wiles & Taft 2010). We cryopreserve embryos or sperm at the earliest stages after deposition and whenever possible with genomic DNA samples and breeding records in order to recover a live colony from earlier generations after extensive breeding periods or whenever undesired mutations are detected.

We have preserved approximately 95% of collected strains as frozen embryos and sperm. A rapidly increasing number of genetically modified strains developed by genome editing have efficiently been cryopreserved by sperm freezing. To protect the archived strains from disasters, the RIKEN BRC has built a back-up facility in the RIKEN Harima branch 700 km away, and every frozen strain has regularly been transferred there since 2007. Moreover, after the Great Eastern Japan Earthquake of 2011, we have installed an in-house water supply system, liquid nitrogen suppliers, and large fuel tanks for an emergency power supply to sufficiently handle a one-week outage to strengthen the security of the main collection in Tsukuba.

## Microbial quality control

Ensuring the reproducibility of animal experiments in life science research is the most important issue for a resource repository. Microbial and genetic QC are two major pillars for the mouse resource repository to ensure reproducible animal experiments. In general, commercial breeders have established global standards and contributed to high-quality animal experiments in Japan. The RIKEN BRC, which regularly receives a number of original developed mouse strains from universities and research institutes, preserves and distributes the mice around Japan and globally, and also plays a critical role in ensuring the microbial quality of the mice made available by eradicating pathogenic agents as listed in Table 2.

**Table 2:** Categorized list of pathogenic microbes to be excluded from the barrier facility at RIKEN BRC

Class	A	B	C	D
Pathogenic agents	*Clostridium piliforme*, Ectromelia virus, Lymphocytic choriomeningitis virus (LCMV), Mouse hepatitis virus (MHV), *Mycoplasma pulmonis*, *Salmonella typhimurium*, Sendai virus (HVJ)	*Filobacterium rodentium*, *Citrobacter rodentium*, *Corynebacterium kutscheri*, Dermatophytes, *Helicobacter bilis*, *Helicobacter hepaticus*, *Pasteurella pneumotropica*, Ectoparasites, Intestinal protozoa, Pinworms, *Salmonella* spp.	*Staphylococcus aureus*, *Pneumocystis murina*, *Pseudomonas aeruginosa*	Lactate dehydrogenase elevating virus (LDHEV), Mouse adenovirus (MAV), Mouse cytomegalovirus (MCMV), Mouse minute virus (MMV), Mouse norovirus (MNV), Mouse parvovirus (MPV), Mouse polyoma virus (Poly), Mouse rotavirus (EDIM), Pneumonia virus of mice (PVM), Reovirus type 3 (Reo 3), Theiler’s mouse encephalomyelitis virus (TMEV)

Mice deposited by universities or institutes are received at the first quarantine facility, where they remain for one night and two days for microbial serology tests and initial external health observations. The mice are subsequently transferred to the second intermediate facility and housed in negative or positive pressure bioBUBBLE Clean Room Units (bioBUBBLE, Inc., Fort Collins, CO) according to the microbial test results. In the second facility, the deposited mice are maintained prior to rederivation procedures, such as breeding for cesarean section or sacrificing sperm donors for in vitro fertilization (IVF) followed by IVF and embryo transfer. After confirming negative results for all the listed pathogenic microbes, mice are introduced into a separate barrier facility, where approximately 10,000 breeding cages can be accommodated.

We have adopted the sentinel mouse program with a specialized protocol for individually ventilated cage units (CLEA Japan, Tokyo, Japan). The program uses dirty bedding sentinels to detect pathogenic microbes. Sentinel mice are exposed to dirty bedding collected from every cage of the unit at regular intervals (Ike et al. 2007). In FY2010, pathogenic agents such as mouse hepatitis virus, *Pasteurella pneumotropica*, and *Helicobacter hepaticus* were detected in 13% of the deposited strains in addition to intestinal protozoa and pinworms in 43% of the strains. Recently, the microbial status of deposited mice has been improved by intensive efforts of research communities to eradicate mouse hepatitis virus and other pathogens, while intestinal protozoa and pinworms have persistently been detected. The RIKEN BRC has also continued to clean-up newly deposited strains for pathogens, such as intestinal pathogens, detected in the mice received from research colonies of universities and institutes.

## Genetic quality control

To set up appropriate genetic QC tests for ensuring reproducibility, we ask all depositor scientists to provide us with precise information including reference publications, primer sequences for genotyping, sequences of vectors and transgenes used for genetic modifications, breeding records, and any other relevant information. If there is any discrepancy between the depositor’s information and our test results, we communicate the results to the depositor and ask for a correction of the supplied information or resending correct mice. We use different genetic QC tests (Nakata et al. 2009, 2021), particularly PCR, depending on various genetic mutations (Table 3). The test results and protocols for each strain are posted in the strain data sheet in the web catalog when tested. When the depositor’s protocol includes methods other than PCR, we design a PCR test so that every user laboratory can easily set up the test based on the information provided by the depositor. Whenever PCR genotyping data from a mutant strain becomes available based on a published paper, we replicate or redesign the PCR genotyping protocol and post it to the web catalog.

**Table 3:** Genetic QC tests conducted at RIKEN BRC

Genetic QC test	Explanations
Transgene-specific PCR	A PCR test that uses transgene-specific primers designed based on information provided by the depositor to detect the promoter and the structural gene driven by the promoter
Targeted gene-specific genotyping PCR	A PCR test that uses targeted gene-specific primers designed based on information provided by the depositor
Detection of marker genes for genetic modifications	PCR tests for TG, BAC-TG, cTG, KO, KI, CRISPR/Cas9, cKO and spontaneous/ENU-induced mutant strains to detect any contamination with other genetically modified mice by using primers for 10 frequently used marker genes, including neo, Pgk-neo, Tk-neo, IRES, lacZ, GFP, Cre, Flp, Puro, and Hyg. (Nakata et al. 2009)
loxP and FRT tests	PCR tests of cTG and cKO mice that examines the fragment length between loxPs or FRTs with primers designed based on information provided by the depositor. Confirmation of structure of conditional strains that carry loxP or FRT flanked alleles. (Nakata et al. 2021)
Detection of BAC-loxP	A PCR test for BAC-TG to detect loxPs derived from BAC vectors
Genetic background test	PCR tests for the genetic background of inbred and wild-derived strains that uses microsatellite or single nucleotide polymorphism (SNP) markers (Mekada et al. 2009, 2015). Test results are posted to the web catalog after testing. Prior to phenotyping at the Japan Mouse Clinic, the strains are subjected to high-speed genetic profiling using the TaqMan assay. (https://ja.brc.riken.jp/lab/jmc/mouse_clinic/en/assistive/index.html)
Sequencing	PCR products are sequenced when the product size is different from the expected size

*TG* transgenic, *BAC-TG* bacterial artificial chromosome-transgenic, *cTG* conditional transgenic, *KO* knockout, *KI* knock-in, *cKO* conditional knockout

Genome editing technology using the CRISPR/Cas9 system (Wang et al. 2013) has facilitated production of genetically modified mouse models across the wider research community and transgenic core facilities around the world. There is a concern, however, that the importance of QC is likely to be overlooked and left behind during rapidly expanding production. Mouse resource organizations around the world that gathered in the INFRAFRONTIER IMPC workshop in Munich agreed to conduct strict QC of mouse resources, including genome-edited mice, in 2014 (Nature editorial 2014). The RIKEN BRC established a policy to accept deposition of genome-edited mouse lines only after confirmation of germline transmission and to not accept founder mice that are likely to be mosaic (Singh et al. 2015; Ayabe et al. 2019) and are, therefore, unsuitable for reproducible experiments. We request the depositor to provide all accurate information, including the results of sequence analyses of mutations induced by genome editing, as listed in Table 4, and preserve the lines by cost-effective sperm freezing. Recently, more attention has been paid to the sequences around the on-target sites to confirm whether the genome was indeed correctly edited as designed and does not contain unintentional genomic changes (Kosicki et al. 2018; Gurumurthy et al. 2019).

**Table 4:** Information required for genetic quality control of CRISPR/Cas9 genome-edited mice

Items	Descriptions
Reference Publications	PubMed ID or DOI
Editing target	Gene or genomic region
Design of editing	Endonuclease (wild-type Cas9, nickase, others), injection reagents (mRNA, DNA, protein), injection methods (Pronuclear, Cytoplasmic, Pronuclear + Cytoplasmic, electroporation, others)
Guide RNA	Sequence and vector IDs
Sequencing results	Wild-type and mutated genome sequences
Detection of the mutation	PCR (primer sequence, PCR condition, fragment size), sequencing, RFLP, TaqMan real-time PCR etc
Off-target analysis	Done or not done
Background strain	Background strain: C57BL/6 N, C57BL/6 J, or others
Generation	Filial or backcross generations
Pedigree	Breeding records and/or pedigree

## Adding value to collected mouse strains

Researchers generally access a specific mouse strain found in publications because the strain has been used by others and proved to be useful through providing relevant information for a particular research purpose. Therefore, information about publications by users is indispensable for enriching the value of each research resource. In addition to direct feedback from users, the RIKEN BRC systematically collects information about users’ publications mainly by using the alert function of Google Scholar and Stanford HighWire and by emails received whenever such publications appear on the website. After manually confirming RBRC strains in publications, we add the paper to the users’ publication list. Such publication information is regularly added to the strain data sheet of the RIKEN BRC Mouse web catalog and then transferred to the NBRP Information Center to update the Research Resource Circulation (RRC) database to enrich the value of NBRP resources (https://rrc.nbrp.jp/projects/1?lang=en).

Phenotypic data for each strain is an important key to evaluating the strain as a potential human disease model or a tool to study disease mechanisms. The Integrated Bioresource Information Division has recently upgraded the mouse web catalog by manually adding 24,490 phenotype annotations to over 4300 strains from publications so that users can find a specific mouse strain based on phenotype ontology terms in addition to gene names (Masuya et al. 2021), thus increasing the findability of the strains. We also promote comprehensive phenotyping of deposited mouse strains by the Japan Mouse Clinic at the RIKEN BRC (Wakana et al. 2009). The broad-based phenotyping platform has been built in collaboration with the International Mouse Phenotyping Consortium (IMPC) to add useful phenotyping data, which should facilitate evaluation of the strain as a potential disease model as demonstrated by the IMPC (Dickinson et al. 2016; Meehan et al. 2017; Birling et al. 2021). Thus far, 69 domestic users have agreed to deposit their 121 mouse strains with phenotypic data generated at the Japan Mouse Clinic, which will be available to the public soon after publication.

Collaborations with the NBRP Genome Information Upgrading Program have contributed to the addition of genome sequence data for wild-derived strains of *Mus musculus*, including the Japanese wild-derived strains MSM and JF1 in the MoG^+^ mouse genomic variation database (https://molossinus.brc.riken.jp/mogplus/#JF1; Takada et al. 2013, 2015). The genome information is essential and useful for future functional studies based on comprehensive phenotyping and genetic modifications of genetically divergent unique mouse strains created by genome editing (Hirose et al. 2017).

## International collaborations

The RIKEN BRC has participated in the International Mouse Strain Resource (IMSR), a one-stop shop database operated by the Jackson Laboratory (Epigg et al. 2015) to register mouse resources developed by Japanese scientists and share those resources with the global biomedical community. Scientists who find interesting strains or queries in the IMSR list can be directed to the holder repository with one click. This function has increased the visibility and accessibility of our mouse strains. By the further collaboration of the IMSR with the Resource Identification Initiative (Bandrowski et al. 2016), our mouse resources are given unique Research Resource Identifiers in addition to repository-specific RBRC numbers, which ensures the ability to identify the exact mouse strains used in publications as well as reproducibility.

Since 2006, mouse repositories and genetics institutes in Asia and Australia have established the Asian Mouse Mutagenesis & Resource Association (AMMRA) to strengthen communications and technical exchanges in the Asia and Australia regions (http://ammra.info/). We have collaborated with major global repository members such as the Jackson Laboratory and the Mutant Mouse Resource and Research Centers in USA, the European Mouse Mutant Archive in Europe, the Centre for Phenogenomics in Canada, and AMMRA members mutually to provide technical assistance in cryo-recovery to local investigators. Moreover, the RIKEN BRC has participated in the IMPC and contributed to the production, preservation, QC, phenotyping, and distribution of knockout mouse resources (Dickinson et al. 2016; Birling et al. 2021). The RIKEN BRC has joined with the IMPC and AMMRA members in the activities of the Global Mouse Models for COVID-19 Consortium and posted our own biological resources, including mouse strains, for respective studies. Thus, our international collaborations and networks function together to facilitate the active use of high-quality mouse resources around the world.

## Conclusion

As progress in decoding the genome of patients continues, the need to verify causative gene candidates underlying genetic diseases in a model organism increases exponentially. Recent genome editing technology enables us to collaborate with clinical scientists in the generation of knock-in mice for variants or mutations found in patients, in addition to the development of null-knockout mice in the same production platform (Lloyd et al. 2020). The Next Generation Human Disease Model Team of the RIKEN BRC has started a new research and development program with the goals of promoting precise mouse modeling of human diseases and contributing to the realization of precision medicine. Advances in genome editing technology will be a major key to the expansion of such mouse resources, while developing technology for use in genome editing may inevitably generate new quality issues that need to be controlled. As a national and international hub of mouse resources and as a critical research resource infrastructure, we will continue expansion of the premier mammalian model organism, mice. We will do this in collaboration with global mouse repositories to make available genetics tools to decipher the molecular mechanisms of health and disease with the highest priority in order to ensure the reproducibility of life science experiments and innovation.
